# A Corticobasal Syndrome Variant of Familial Creutzfeldt-Jakob Disease with Stroke-Like Onset

**DOI:** 10.1155/2016/4167391

**Published:** 2016-10-10

**Authors:** Ján Necpál, Martin Stelzer, Silvia Koščová, Michal Patarák

**Affiliations:** ^1^Department of Neurology, Zvolen Hospital, Kuzmányho nábrežie 28, 960 01 Zvolen, Slovakia; ^2^Department of Prion Diseases, Slovak Medical University, Limbová 14, 833 03 Bratislava, Slovakia; ^3^Department of Psychiatry, Roosevelt Hospital, Cesta k nemocnici 1, 975 56 Banská Bystrica, Slovakia

## Abstract

Creutzfeldt-Jakob disease (CJD) is an untreatable rare human prion disease characterized by rapidly progressive dementia along with various neurological features, including myoclonus and sometimes other movement disorders. The clinical course is typically insidious and rapid, leading to an early death. In general, the most common form is sporadic CJD; however, Slovakia is typical for a high percentage of genetic cases. We present an unusual case report of a 65-year-old man with a sudden, stroke-like onset of motor aphasia with right-sided levodopa unresponsive parkinsonism, alien hand, and other characteristic features of corticobasal syndrome (CBS), with rapid deterioration and death on the 32nd day of the disease. Various neurodegenerative disorders are manifested with CBS as a clinical phenotype, including corticobasal degeneration (CBD), progressive supranuclear palsy, Alzheimer's disease, and CJD. In our patient, mutation E200K and M129M polymorphism of the PRNP gene and typical immunohistochemical findings pointed to a diagnosis of CJD. The patient's mother died of CJD many years ago. Several CBS-CJD cases were described, but the atypical stroke-like onset of CBS-CJD, an extremely rare presentation of CJD, makes our case unique worldwide.

## 1. Introduction

Creutzfeldt-Jakob disease (CJD) is a rare human prion disease with annual incidence of 1–1.5/million inhabitants, characterized by rapidly progressive dementia, myoclonus, ataxia, visual symptoms, pyramidal and extrapyramidal disturbance, akinetic mutism, and early death [1]. Although the majority of cases are sporadic, genetic forms with CJD-specific mutations also occur. Slovakia is characterized by an unusually high percentage (65–75%) of genetic cases, with mutation E200K of the prion protein gene being the most frequent [2]. Apart from myoclonus, the disease rarely presents with other movement disorders, such as chorea, tremor, dystonia, and parkinsonism, including a progressive supranuclear palsy-like and corticobasal syndrome phenotype [3].

## 2. Case Presentation

**Figure 1:**
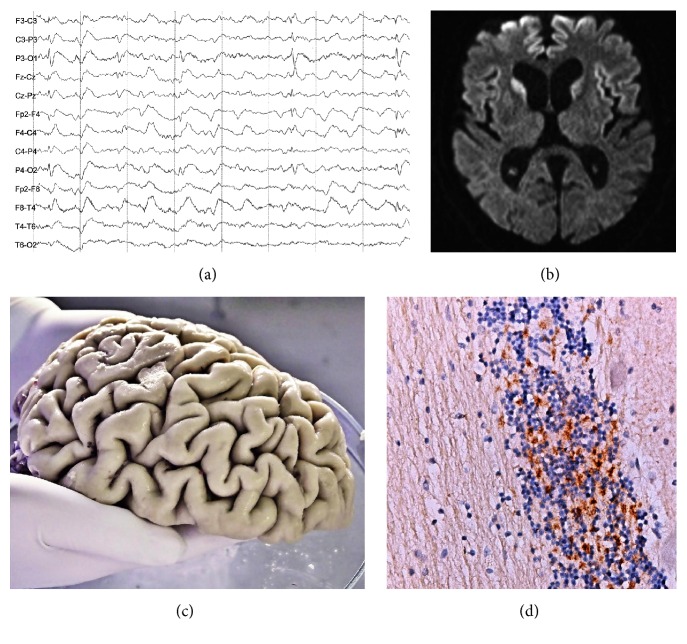
Antemortem and postmortem findings in an index case. (a) An EEG with pseudoperiodic generalized triphasic slow waves on the 23rd day of the disease. (b) Axial brain DWI shows hyperintense areas in bilateral caudate nuclei, bilateral frontal, and left insular cortex. (c) Macroscopic view of the left frontal lobe atrophy. (d) Immunohistochemical confirmation of prion protein with 6H4 monoclonal antibodies (granular layer of the cerebellar cortex, 400x zoom).

We present a 65-year-old man with a history of hypertension, coronary artery disease, and diabetes admitted to the hospital due to the sudden onset of nonfluent aphasia as a sign of possible stroke. The brain CT with angiography, carotid angiogram, EKG, and echocardiogram were all unremarkable. Logopaedic and psychological assessment revealed motor aphasia, bradylalia, marked response latency, apraxia, acalculia, and alexia with agraphia. The Standardized Mini-Mental State Examination score was 14/30. An EEG on the 6th and 13th day after onset was normal. Over the next few days, the patient developed a shuffling and apractic gait, levodopa unresponsive predominantly right-sided hemiparkinsonism with slight action dystonia of the right hand, upgoing toes, ideomotoric apraxia with forced grasping, mirror movements, alien hand, and positive applause sign—typical features of corticobasal syndrome (CBS). He would sometimes hold his right upper limb for several minutes in an odd-looking posture with extension, levitation, and grasping (probably a manifestation of alien hand syndrome). There were no speech, motor, or cognition problems before the clinical onset of disease. Some members of the patient's family had died of some rapidly progressive neurological disorder, and his mother had definite CJD. Routine biochemistry, ANA and ANCA antibodies, thyroglobulin and thyroid peroxidase antibodies, tests for HIV and syphilis, virology, and onconeural and autoimmune encephalitis antibodies were all negative. There was mild hyperproteinorhachia (0,63 g/L) and increased total tau protein but negative 14-3-3 protein in the cerebrospinal fluid. The EEG taken on the 23rd day displayed marked diffuse slowing with pseudoperiodic generalized triphasic slow wave pattern (Figure 1(a)). Brain MRI performed on the 23rd day revealed slight symmetrical frontoparietal atrophy and high signal in both caudate nuclei and the bilateral frontal and left insular cortex on diffuse-weighted images (DWI) sequence, with no signs of CBS (Figure 1(b)). There were no clear cortical T2 changes. FLAIR sequence was unfortunately not performed. Diagnosis of probable CJD was then made. The disease rapidly progressed into akinetic mutism, with generalized hypertonia and finally death on the 32nd day of the disease. Mutation E200K and M129M polymorphism of the PRNP gene were identified. An autopsy revealed macroscopically a mild diffuse atrophy of the frontal, paracentral, and cerebellar cortex (Figure 1(c)). The typical histopathological triad of spongiosis, neuronal loss, and astrogliosis, as well as immunohistochemically detected pathological prion protein, confirmed the diagnosis of definite CJD (Figure 1(d)).

## 3. Discussion

To date, 39 cases worldwide with the CBS presentation of CJD (CBS-CJD) have been published [4]. Lee et al. reported the clinical characteristics of 20 such autopsy cases with extremely low annual incidence of this condition (0.014/million cases). Mean age at onset was 66 years. They presented with limb apraxia, alien hand phenomenon, dysphasia, cortical sensory loss, myoclonus, dystonia, rigidity, and pyramidal disturbance—classic CBS signs. The only criterion clinically distinguishable between pathologically confirmed CBD with CBS and CBS-CJD was the rapidity of disease progression. Median time from disease onset to death was 68 months in CBD and only 5 months in CBS-CJD [5]. Typical MRI changes, periodic sharp waves on the EEG, and 14-3-3 protein positivity may be helpful for diagnosis (however, they are not specific and may be absent) and along with dementia are otherwise cardinal features of CJD [4, 5]. The presence of true dementia in our patient remains questionable, because cognitive testing was probably influenced by aphasia and apathy. The duration of the illness from onset to death was only 32 days. The clinical course itself was very similar to the reported case of a 73-year-old woman with acute CBS-CJD of four weeks' duration [6], except for being familial in our patient. There is only one reported case of CBS-CJD in the context to familial CJD with the mutation M232R [7]. An extraordinary sudden stroke-like onset of the disease contrasting to more typical gradual onset of CJD, a CBD-look-alike presentation, and finally the familial occurrence of CJD all appeared in one person; therefore, we consider this case as unique and worth reporting. It should serve as a clue for clinicians to keep in mind that rapidly progressive atypical parkinsonian syndromes may masquerade as CJD, and genetic testing for CJD-specific mutations may significantly contribute to differential diagnosis of neurodegenerative disorders.
